# First Annual Report for Robot‐Assisted Surgery Based on the National Clinical Database 2019 in Japan: Report on Three Major Gastrointestinal Fields

**DOI:** 10.1111/ases.70220

**Published:** 2026-01-22

**Authors:** Ichiro Takemasa, Hiroyuki Yamamoto, Tatsuto Nishigori, Takeo Fujita, Tomoki Makino, Yusuke Taniyama, Masanori Terashima, Masanori Tokunaga, Takatoshi Matsuyama, Tomohiro Yamaguchi, Noriko Iwata, Hidetoshi Katsuno, Koichi Suda, Yusuke Kinugasa, Kazutaka Obama, Takashi Kamei, Ichiro Uyama, Masahiko Watanabe, Yoshiharu Sakai, Yuko Kitagawa

**Affiliations:** ^1^ Department of Gastroenterological Surgery Osaka International Medical and Science Center, Osaka Keisatsu Hospital Osaka Japan; ^2^ Department of Healthcare Quality Assessment, Graduate School of Medicine The University of Tokyo Tokyo Japan; ^3^ Department of Surgery Kyoto University Kyoto Japan; ^4^ Department of Esophageal Surgery National Cancer Center Hospital East Kashiwa Japan; ^5^ Department of Gastroenterological Surgery Osaka University Graduate School of Medicine Suita Japan; ^6^ Department of Surgery Tohoku University Graduate School of Medicine Sendai Japan; ^7^ Division of Gastric Surgery Shizuoka Cancer Center Shizuoka Japan; ^8^ Department of Gastrointestinal Surgery Institute of Science Tokyo Tokyo Japan; ^9^ Department of Digestive Tract and General Surgery, Saitama Medical Center Saitama Medical University Saitama Japan; ^10^ Department of Gastroenterological Surgery Cancer Institute Hospital of Japanese Foundation for Cancer Research Tokyo Japan; ^11^ Department of Surgery Edogawa Hospital Tokyo Japan; ^12^ Department of Surgery Fujita Health University School of Medicine Okazaki Medical Center Toyoake Japan; ^13^ Divisions of GI & HPB, Department of Surgery Fujita Health University Toyoake Japan; ^14^ Department of Advanced Robotic and Endoscopic Surgery, School of Medicine Fujita Health University Toyoake Japan; ^15^ Department of Surgery Kitasato University Kitasato Institute Hospital Tokyo Japan; ^16^ Director, Red Cross Hospital Osaka Osaka Japan; ^17^ Department of Surgery Keio University School of Medicine Tokyo Japan

**Keywords:** annual report, gastroenterological surgery, National Clinical Database, registry, robot‐assisted surgery, surgical outcome

## Abstract

**Aim:**

The adoption of robot‐assisted surgery (RAS) in Japan has progressed significantly since its initial approval in 2009. RAS gradually expanded into various surgical fields with 35 procedures now covered under Japan's national health insurance. This study provides an inaugural assessment of RAS outcomes for seven digestive procedures introduced in 2018.

**Methods:**

The Japanese Society for Endoscopic Surgery working group established an RAS registry integrating data from the National Clinical Database and additional RAS‐specific records. The analysis focused on three major gastrointestinal fields: the esophagus, stomach, and rectum.

**Results:**

In 2019, 530 esophagectomies, 2295 gastrectomies, and 3269 proctectomies were performed. RAS for these procedures was characterized by relatively long operative times, low intraoperative blood loss, and very low conversion rates to open surgery (< 1%). Postoperative morbidity rates Grade IIIa or higher were 23.2% for esophagectomy, 4.9% for gastrectomy, and 9.4% for proctectomy. Length of postoperative hospital stay correlated with morbidity, though readmission (1.3%–3.1%) and postoperative mortality rates (0.3%–0.6%) remained low. The early nationwide implementation of RAS in Japan was marked by a high surgeon qualification rate (98.9%) and meticulous case selection; the DVSS Xi model accounted for 66.3% of robotic platforms used.

**Conclusion:**

These findings underscore the need for ongoing surveillance and data‐driven evaluation to ensure safe and effective implementation of RAS. Future longitudinal analyses will refine surgical quality, optimize resource allocation, and advance minimally invasive techniques. This study highlights the transformative potential of RAS in Japanese surgical practice and its alignment with global trends.

## 1 Introduction

Robot‐assisted surgery (RAS) using the da Vinci Surgical System (DVSS) has been widely adopted in Japan since its approval by the Pharmaceuticals and Medical Devices Agency (PMDA) in 2009. Initially applied in urology, RAS gained its first insurance reimbursement in 2012 for robot‐assisted radical prostatectomy, marking the beginning of its expansion into other surgical fields.

A paradigm shift occurred in 2016 with reimbursement of robot‐assisted laparoscopic partial nephrectomy, leading to the widespread adoption of RAS in gastrointestinal, thoracic, and gynecological surgeries. By 2018, insurance coverage extended to 12 additional procedures, including gastric, esophageal, rectal, and lung cancer treatment. Subsequent expansions in 2020 and 2022 included pancreatic, colon, and liver cancer procedures.

Initially, RAS was reimbursed at the same rate as laparoscopic and thoracoscopic surgeries due to insufficient evidence of superiority. However, advancements in clinical research, including a multi‐institutional, single‐arm prospective phase II clinical trial for gastric cancer [1], led to additional reimbursement in 2022. As of 2024, insurance coverage encompasses 35 procedures across six surgical fields: respiratory, cardiac, digestive, urology, gynecology, and orthopedics.

Despite its undeniable advantages, minimally invasive approaches such as RAS do not eliminate the possibility of adverse events [2]. The rapid increase in RAS cases has been accompanied by a rise in reported complications [3], highlighting the need for a structured, cross‐disciplinary infrastructure [4]. Given the high cost of RAS, establishing additional surgical fees and evaluating its cost‐effectiveness remain critical considerations [5, 6].

To date, no national, comprehensive data specific to RAS have been available to provide a complete overview of its overall status. Therefore, to thoroughly assess the current state and impact of RAS in Japan, the Japanese Society for Endoscopic Surgery (JSES) has incorporated RAS‐specific data into the National Clinical Database (NCD), creating a large‐scale, real‐world RAS database. This report serves as the first annual summary, offering valuable insights into the current state and future direction of RAS in Japan.

## 2 Materials and Methods

This study focuses on seven digestive tract surgical procedures that became eligible for reimbursement in 2018: esophagectomy, proximal gastrectomy (PG), distal gastrectomy (DG), total gastrectomy (TG), anterior resection of the rectum (AR), intrasphincteric resection (ISR), and abdominoperineal resection (APR). These procedures span three major gastrointestinal fields: esophagus, stomach, and rectum. To establish a unified framework for the RAS registry, JSES working groups standardized key data points across the three fields based on the NCD, following the “Curriculum for Training in Gastroenterological Surgery” by the Japanese Society of Gastroenterological Surgery (JSGS).

### 2.1 Data Collected

A total of 28 data items were included in the analysis, covering demographic, surgical, and postoperative outcomes:

- **Demographics:** Gender, age, and preoperative treatment status.
- **Tumor findings:** Pathological stage based on depth of tumor invasion, extent of lymph node metastasis, and distant metastasis.
- **Surgical details:** Procedure type, operation time, intraoperative blood loss, extent of lymph node dissection, R0 resection, intraoperative blood transfusions, intraoperative adverse events, conversion to open surgery, and robotic system used.
- **Surgeon certification:** Qualification under the Endoscopic Surgical Skill Qualification (ESSQ) System of JSES.
- **Postoperative outcomes:** Morbidity grade, length of postoperative hospital stay (LPHS), readmission within 30 days, and postoperative mortality.
- **Procedure‐specific items:** Thoracoscopy, mediastinoscopy, robotic approach in esophagectomy and lateral lymph node dissection in proctectomy.
- **Facility metrics:** Number of institutions performing RAS.

Postoperative complications were classified into six levels using the Clavien–Dindo (C–D) method [7], with severe complications defined as Grade III or higher.

### 2.2 Study Period and Statistical Analysis

Given the short registration period in 2018, 2019 was designated as the first study year. Statistical analyses were conducted by an NCD statistician. Data handling and statistical analyses were performed using STATA 16 software (STATA Corp., TX, USA).

### 2.3 Important Considerations

1. **Simultaneous procedures:** Cases involving multiple surgeries were counted for all relevant procedures.
2. **Postoperative mortality:** Postoperative mortality includes deaths during hospitalization (up to 90 days post‐surgery) and deaths within 30 days post‐discharge. This composite definition follows the current standard reporting format used in NCD‐based annual reports for gastroenterological surgery and was adopted to ensure consistency and enable longitudinal comparison.

The present study was approved by the Ethics and Conflict of Interest Committee of the NCD. This standardized and comprehensive approach enables the evaluation of RAS outcomes across multiple procedures and institutions, laying the foundation for further analyses and improvements in surgical practices.

## 3 Results

### 3.1 Number of RAS Cases by Surgical Procedure

The number of cases and surgical procedures were as follows: esophagectomy for esophageal cancer, 530 cases; gastrectomy for gastric cancer, 2295 cases (DG 1682 cases, TG 323 cases, PG 290 cases); and proctectomy for rectal cancer, 3269 cases (AR 2590 cases, ISR 231 cases, APR 356 cases). For esophageal cancer, subtotal resection accounted for 97.5% of cases. Among thoracic approaches, the transthoracic approach was utilized in 92.5% of cases, while the transhiatal approach was used in 6.4%. In abdominal approaches, RAS was performed in 36.8%, while laparoscopic procedures were used in 48.9%, together comprising approximately half of the cases (Table S1). The male‐to‐female ratio across all procedures was approximately 3:1, skewed toward males, though the degree of skewness varied by organ (Table 1). The proportion of patients aged under 60 was similar across procedures, while the percentage of patients aged 80 or older was higher for gastrectomy (13.6%) and proctectomy (10.3%) compared to esophagectomy (4.7%) (Table 1).

**TABLE 1:** Patient characteristics.

		Esophagectomy	Gastrectomy				Proctectomy			
		All procedures	All procedures	DG	TG	PG	All procedures	AR	ISR	APR
		*N* = 530	*N* = 2295	*N* = 1682	*N* = 323	*N* = 290	*N* = 3269	*N* = 2590	*N* = 231	*N* = 356
Sex	Female	118 (22.3%)	763 (33.2%)	588 (35.0%)	100 (31.0%)	75 (25.9%)	1151 (35.2%)	921 (35.6%)	70 (30.3%)	137 (38.5%)
	Male	412 (77.7%)	1532 (66.8%)	1094 (65.0%)	223 (69.0%)	215 (74.1%)	2118 (64.8%)	1669 (64.4%)	161 (69.7%)	219 (61.5%)
Age	≤ 59	123 (23.2%)	471 (20.5%)	57 (19.7%)	344 (20.5%)	70 (21.7%)	930 (28.4%)	94 (40.7%)	763 (29.5%)	58 (16.3%)
	60–64	91 (17.2%)	247 (10.8%)	29 (10.0%)	180 (10.7%)	38 (11.8%)	413 (12.6%)	29 (12.6%)	334 (12.9%)	45 (12.6%)
	65–69	124 (23.4%)	376 (16.4%)	43 (14.8%)	275 (16.3%)	58 (18.0%)	576 (17.6%)	42 (18.2%)	465 (18.0%)	55 (15.4%)
	70–74	106 (20.0%)	496 (21.6%)	51 (17.6%)	367 (21.8%)	78 (24.1%)	581 (17.8%)	40 (17.3%)	464 (17.9%)	70 (19.7%)
	75–79	61 (11.5%)	392 (17.1%)	55 (19.0%)	286 (17.0%)	51 (15.8%)	432 (13.2%)	19 (8.2%)	330 (12.7%)	62 (17.4%)
	≥ 80	25 (4.7%)	313 (13.6%)	55 (19.0%)	230 (13.7%)	28 (8.7%)	337 (10.3%)	7 (3.0%)	234 (9.0%)	66 (18.5%)
Preoperative treatment	ESD	30 (5.7%)	237 (10.3%)	186 (11.1%)	14 (4.3%)	37 (12.8%)	N/A	N/A	N/A	N/A
	Chemotherapy	254 (47.9%)	71 (3.1%)	32 (1.9%)	31 (9.6%)	8 (2.8%)	252 (7.7%)	153 (5.9%)	44 (19.0%)	41 (11.5%)
	Chemoradiation	24 (4.5%)	N/A	N/A	N/A	N/A	191 (5.8%)	86 (3.3%)	27 (11.7%)	72 (20.2%)
	Radiation	0	N/A	N/A	N/A	N/A	21 (0.6%)	8 (0.3%)	1 (0.4%)	12 (3.4%)
	None	207 (39.1%)	1898 (82.7%)	1400 (83.2%)	263 (81.4%)	235 (81.0%)	2754 (84.2%)	2302 (88.9%)	157 (68.0%)	226 (63.5%)
	Missing	15 (2.8%)	89 (3.9%)	64 (3.8%)	15 (4.6%)	10 (3.4%)	51 (1.6%)	41 (1.6%)	2 (0.9%)	5 (1.4%)
pStage	≤ Stage I	234 (44.2%)	1460 (63.6%)	1142 (67.9%)	133 (41.2%)	185 (63.8%)	1109 (33.9%)	904 (34.9%)	99 (42.9%)	81 (22.8%)
	Stage II	100 (18.9%)	425 (18.5%)	301 (17.9%)	75 (23.2%)	49 (16.9%)	739 (22.6%)	581 (22.4%)	42 (18.2%)	94 (26.4%)
	Stage III	109 (20.6%)	302 (13.2%)	182 (10.8%)	92 (28.5%)	28 (9.7%)	989 (30.3%)	815 (31.5%)	58 (25.1%)	90 (25.3%)
	Stage IV	56 (10.6%)	52 (2.3%)	30 (1.8%)	16 (5.0%)	6 (2.1%)	248 (7.6%)	183 (7.1%)	8 (3.5%)	40 (11.2%)
	Stage X	22 (4.2%)	56 (2.4%)	27 (1.6%)	7 (2.2%)	22 (7.6%)	47 (1.4%)	33 (1.3%)	5 (2.2%)	7 (2.0%)
	Others	9 (1.7%)	N/A	N/A	N/A	N/A	137 (4.2%)	74 (2.9%)	19 (8.2%)	44 (12.4%)

*Note:* Distal gastrectomy included pylorus‐preserving gastrectomy (*N* = 48). All procedures of esophagectomy = Subtotal esophagectomy + Lower esophagectomy + Pharyngolaryngo‐esophagectomy + Others. All procedures of gastrectomy = DG + TG + PG. All procedures of proctectomy = AR + ISR + APR + Hartman + TAMIS.

Abbreviations: APR, abdominoperineal resection; AR, anterior resection; DG, distal gastrectomy; ESD, endoscopic submucosal dissection; ISR, intersphincteric resection; N/A, not applicable; PG, proximal gastrectomy; TG, total gastrectomy.

### 3.2 Number of Facilities Performing RAS and Types of Equipment Used

**FIGURE 1:**
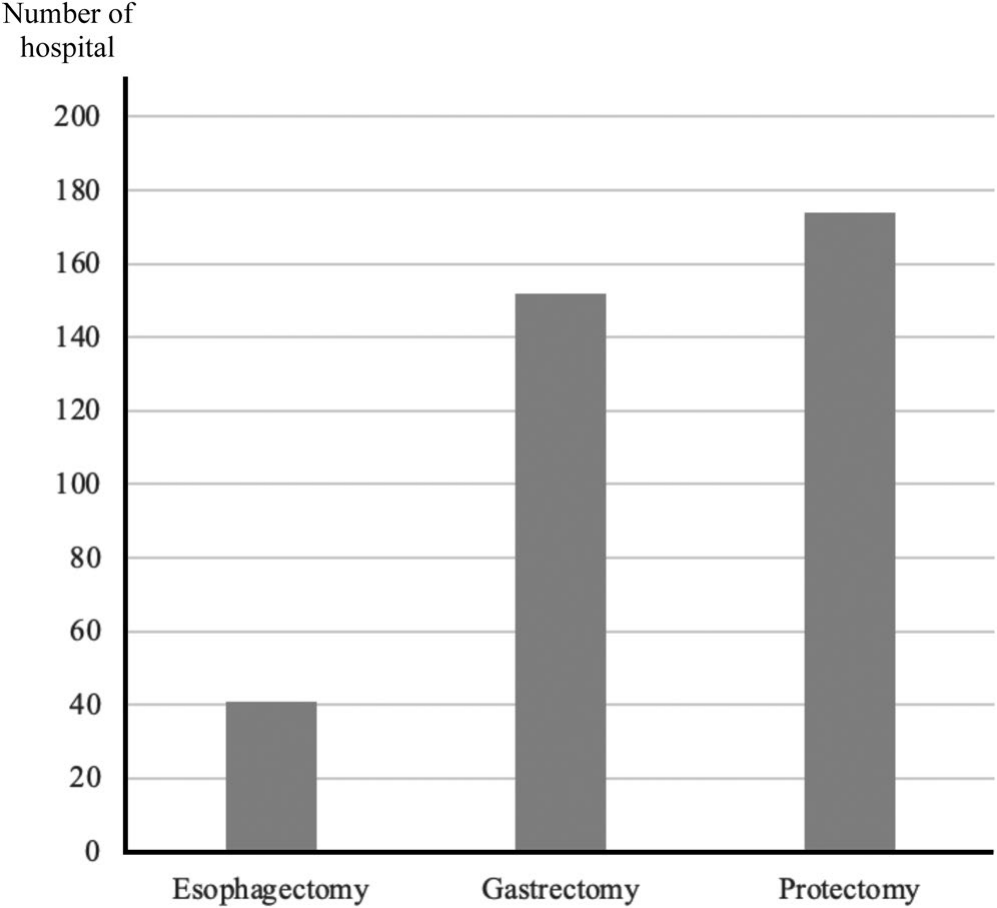
Number of facilities in Japan performing RAS by procedure in 2019.

In 2019, 41 facilities performed robot‐assisted esophagectomy, 152 performed gastrectomy, and 174 performed proctectomy (Figure 1), representing only 0.8%–3.5% of the approximately 5000 facilities participating in the NCD registry. Notably, 98.9% of RAS procedures were performed by ESSQ‐qualified surgeons of JSES (Table 2). The types of surgical robots used were S (1.4%), Si (29.9%), X (2.1%), and Xi (66.3%) among the DVSS models (Table 2).

**TABLE 2:** Surgical details.

		Esophagectomy	Gastrectomy				Proctectomy			
		All procedures	All procedures	DG	TG	PG	All procedures	AR	ISR	APR
		*N* = 530	*N* = 2295	*N* = 1682	*N* = 323	*N* = 290	*N* = 3269	*N* = 2590	*N* = 231	*N* = 356
ESSQ‐qualified surgeon	Yes	509 (96.0%)	2280 (99.3%)	1670 (99.3%)	320 (99.1%)	290 (100.0%)	3236 (99.0%)	2558 (98.8%)	230 (99.6%)	356 (100.0%)
	No	17 (3.2%)	15 (0.7%)	12 (0.7%)	3 (0.9%)	0 (0.0%)	33 (1.0%)	32 (1.2%)	1 (0.4%)	0
	Missing	4 (0.8%)	0	0	0	0	0	0	0	0
Type of surgical robot	DVSS S	13 (2.5%)	50 (2.2%)	37 (2.2%)	6 (1.9%)	7 (2.4%)	22 (0.7%)	19 (0.7%)	1 (0.4%)	1 (0.3%)
	DVSS Si	108 (20.4%)	731 (31.9%)	542 (32.2%)	97 (30.0%)	92 (31.7%)	986 (30.2%)	809 (31.2%)	46 (19.9%)	102 (28.7%)
	DVSS X	1 (0.2%)	44 (1.9%)	35 (2.1%)	3 (0.9%)	6 (2.1%)	85 (2.6%)	74 (2.9%)	2 (0.9%)	6 (1.7%)
	DVSS Xi	404 (76.2%)	1468 (64.0%)	1067 (63.4%)	216 (66.9%)	185 (63.8%)	2169 (66.4%)	1684 (65.0%)	182 (78.8%)	245 (68.8%)
	Missing	4 (0.8%)	2 (0.1%)	1 (0.1%)	1 (0.3%)	0	7 (0.2%)	4 (0.2%)	0 (0.0%)	2 (0.6%)
Median operation time	min	273 (215–330)*	358 (298–430)	340 (287–407)	433 (365–535)	395 (342–458)	348 (272–440)	332 (261–419)	423 (351–543)	411 (335–537)
Median blood loss	ml	124 (60–238)	20 (5–50)	15 (5–43)	32.5 (10–98)	40 (10–116)	15 (3–50)	10 (0–36)	50 (15–120)	80 (30–198.5)
Lymph node dissection	D0	5 (0.9%)	11 (0.5%)	6 (0.4%)	0	5 (1.7%)	15 (0.5%)	5 (2.2%)	5 (0.2%)	5 (1.4%)
	D1	8 (1.5%)	1375 (59.9%)	982 (58.4%)	118 (36.5%)	275 (94.8%)	8 (0.2%)	0	2 (0.1%)	5 (1.4%)
	D2	296 (55.8%)	909 (39.6%)	694 (41.3%)	205 (63.5%)	10 (3.4%)	589 (18.0%)	54 (23.4%)	462 (17.8%)	55 (15.4%)
	Missing	12 (2.3%)	0	0	0	0	0	0	0	0
	D3	209 (39.4%)	N/A	N/A	N/A	N/A	2657 (81.3%)	172 (74.5%)	2121 (81.9%)	291 (81.7%)
Intraoperative transfusion		49 (9.2%)	38 (1.7%)	22 (1.3%)	8 (2.5%)	8 (2.8%)	49 (1.5%)	30 (1.2%)	2 (0.9%)	13 (3.7%)
Intraoperative adverse events		16 (3.0%)	24 (1.0%)	15 (0.9%)	5 (1.5%)	4 (1.4%)	44 (1.3%)	22 (0.8%)	17 (7.4%)	3 (0.8%)
Open conversion		2 (0.4%)	16 (0.7%)	6 (0.4%)	8 (2.5%)	2 (0.7%)	12 (0.4%)	9 (0.3%)	0	1 (0.3%)
R0 resection		487 (91.9%)	2203 (96.0%)	1631 (97.0%)	303 (93.8%)	269 (92.8%)	3073 (94.0%)	2478 (95.7%)	218 (94.4%)	302 (84.8%)

*Note:* All procedures of esophagectomy = Subtotal esophagectomy + Lower esophagectomy + Pharyngolaryngo‐esophagectomy + Others. All procedures of gastrectomy = DG + TG + PG. All procedures of proctectomy = AR + ISR + APR + Hartman + TAMIS. All procedures of proctectomy = AR + ISR + APR + Hartman + TAMIS.

Abbreviations: APR, abdominoperineal resection; AR, anterior resection; DG, distal gastrectomy; DVSS, da Vinci surgical system; ESSQ, the Endoscopic Surgical Skill Qualification System of the Japanese Society of Endoscopic Surgery; ISR, intersphincteric resection; N/A, not applicable; PG, proximal gastrectomy; TAMIS, transanal minimally invasive surgery; TG, total gastrectomy.

* Operation time for thoracoscopy.

### 3.3 Operative Outcomes of RAS

Lymph node dissection was comprehensive, with D2 or higher dissection in 95.3% of esophageal cancer cases, D1 or higher in 99.5% of gastric cancer cases, and D2 or higher in 99.3% of rectal cancer cases (Table 2). Lateral lymph node dissection (LLND) for advanced rectal cancer was performed in 13.2% of proctectomy cases overall, 24.7% for ISR, and 30.6% for APR (Table S2). The R0 resection rates were 91.9% for esophagectomy, 96.0% for gastrectomy, and 94.0% for proctectomy (Table 2). Most patients underwent radical resection. Median operation time and blood loss were 273 min and 124 mL for the thoracic portion of esophagectomy, 358 min and 20 mL for gastrectomy, and 348 min and 15 mL for proctectomy, respectively. Although the rate of conversion to open surgery for TG was relatively high at 2.5%, it remained below 1% across all three major fields when considered as a whole (Table 2). Overall, RAS in 2019 was characterized by relatively long operative times in the context of low blood loss and very low conversion rates.

### 3.4 Adverse Events of RAS

**FIGURE 2:**
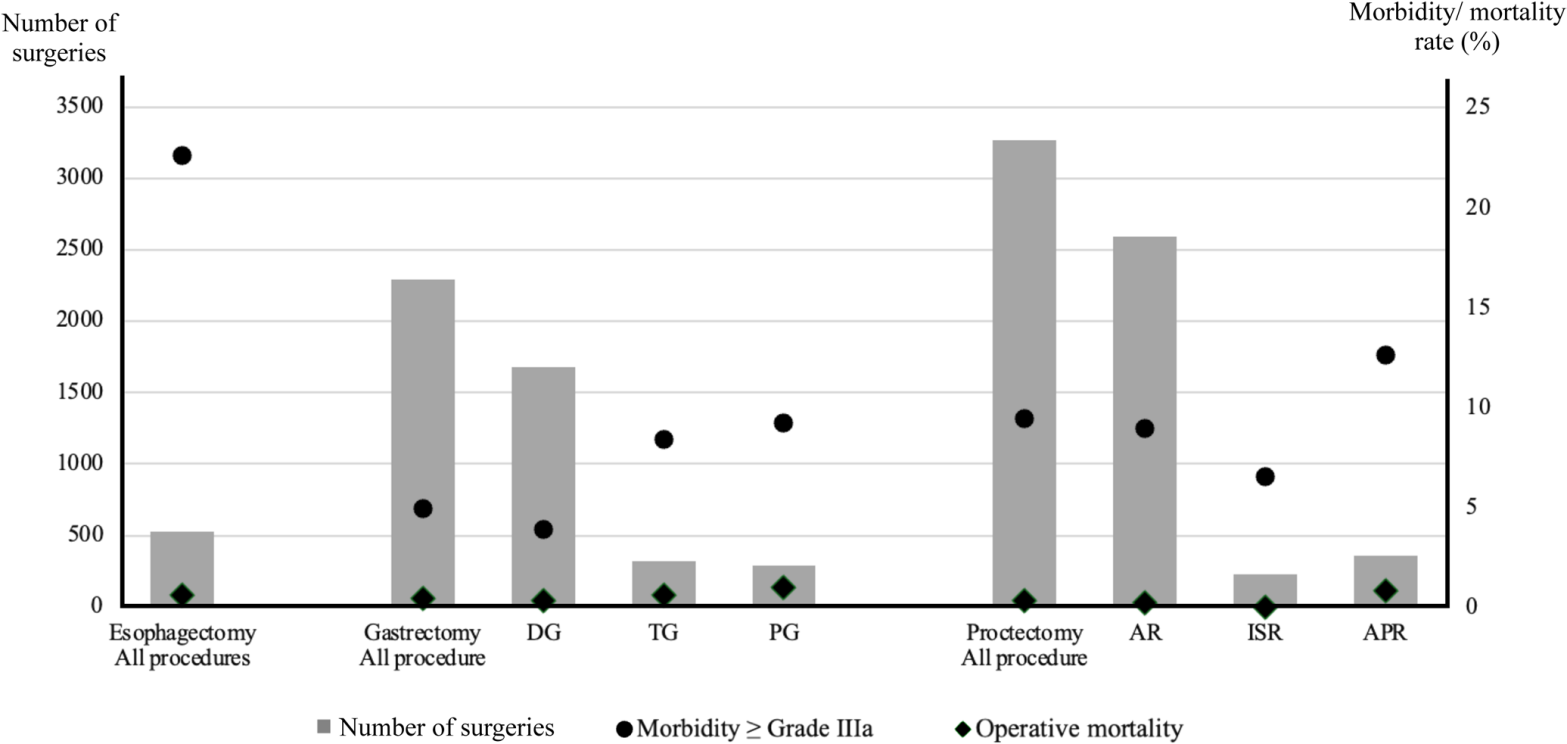
Adverse events in RAS by procedure in Japan in 2019.

Postoperative morbidity varied significantly by procedure. Rates of Grade II or higher and Grade IIIa or higher morbidity were 43.4% and 23.2% for esophagectomy, 12.2% and 4.9% for gastrectomy, and 20.9% and 9.4% for proctectomy, respectively. For gastrectomy, Grade IIIa or higher morbidity rates were 3.9%, 8.4%, and 9.2% for DG, TG, and PG, and for proctectomy, 8.9%, 6.5%, and 12.6% for AR, ISR, and APR, respectively. These rates were substantially higher for esophagectomy than for rectal and gastric procedures (Table 3, Figure 2). Intraoperative adverse events occurred in 3.0% of esophagectomy cases, 1.0% of gastrectomy cases, and 1.3% of proctectomy cases. Intraoperative pulmonary‐related events were observed in six esophagectomy cases (1.1%).

**TABLE 3:** Postoperative outcomes.

	Esophagectomy	Gastrectomy				Proctectomy			
	All procedures	All procedures	DG	TG	PG	All procedures	AR	ISR	APR
	*N* = 530	*N* = 2295	*N* = 1682	*N* = 323	*N* = 290	*N* = 3269	*N* = 2590	*N* = 231	*N* = 356
Morbidity ≥ Grade II	230 (43.4%)	280 (12.2%)	175 (10.4%)	58 (18.0%)	47 (16.2%)	684 (20.9%)	493 (19.0%)	49 (21.2%)	111 (31.2%)
Morbidity ≥ Grade IIIa	123 (23.2%)	113 (4.9%)	65 (3.9%)	27 (8.4%)	21 (9.2%)	306 (9.4%)	230 (8.9%)	15 (6.5%)	45 (12.6%)
LPHS	23 (17–32)	10 [8, 9, 10, 11, 12, 13]	10 (8–12)	11 (9–14)	11 (9–15)	12 (9–18)	12 (9–16)	15 (11–20)	17 (13–23)
Readmission within 30 days after surgery	7 (1.3%)	46 (2.0%)	36 (2.1%)	5 (1.5%)	5 (1.7%)	101 (3.1%)	83 (3.2%)	4 (1.7%)	11 (3.1%)
Postoperative mortality*	3 (0.6%)	10 (0.4%)	5 (0.3%)	2 (0.6%)	3 (1.0%)	11 (0.3%)	6 (0.2%)	0 (0.0%)	3 (0.8%)

*Note:* Distal gastrectomy included pylorus‐preserving gastrectomy (*N* = 48). All procedures of esophagectomy = Subtotal esophagectomy + Lower esophagectomy + Pharyngolaryngo‐esophagectomy + Others. All procedures of gastrectomy = DG + TG + PG. All procedures of proctectomy = AR + ISR + APR + Hartman + TAMIS.

Abbreviations: APR, abdominoperineal resection; AR, anterior resection; DG, distal gastrectomy; ISR, intersphincteric resection; LHPS, length of postoperative hospital stay; PG, proximal gastrectomy; TG, total gastrectomy.

* Postoperative mortality includes deaths during hospitalization (up to 90 days post‐surgery) and deaths within 30 days post‐discharge.

### 3.5 Short‐Term Outcomes of RAS

LPHS increased in proportion to the rate of postoperative morbidity, with esophagectomy at 23 days, gastrectomy at 10 days, and proctectomy at 12 days. On the other hand, readmission rates within 30 days after surgery were low, ranging from 1.3% to 3.1%, and postoperative mortality rates remained low, ranging from 0% to 1.0% across all procedures. Among the gastrectomy procedures, PG had the highest postoperative mortality rate (Table 3).

## 4 Discussion

Fifteen years after its introduction, RAS has become pivotal in Japanese surgical practice, particularly in cancer treatment across various specialties. However, its systematic and safe integration with existing laparoscopic and thoracoscopic techniques remains essential. Robust evidence is critical for evaluating surgical treatments, and real‐world data are increasingly recognized as a valuable tool for overcoming the challenges inherent in prospective randomized controlled trials (RCTs) [8]. While RCTs remain the gold standard, they are not always feasible due to factors such as variations in surgeon expertise, evolving technology, timing of new equipment adoption, statistical variability, and low patient consent acquisition rates [9]. Addressing these challenges underscores the growing importance of real‐world data for generating actionable insights [10].

Since its establishment in 2010, the NCD has grown to become a cornerstone of surgical quality management in Japan [11]. Five additional societies, including JSES, have since joined. As of April 2024, 5679 facilities and 14 920 departments participate in the NCD, registering over 2.63 million cases annually, with a cumulative total of 28.48 million cases over 15 years [12]. Covering over 95% of relevant surgeries, the NCD stands out for its scale and quality [13]. It serves as a critical resource for advancing research, enhancing precision medicine, and promoting transparency in surgical outcomes. By publishing nationwide data through annual reports, it contributes to the evaluation and improvement of medical care, supporting academic research, medical safety, and cost management [14].

In gastroenterological surgery, the NCD has played a pivotal role in establishing consensus. Since 2011, JSGS has incorporated the NCD into annual reports, initially focusing on single‐year metrics but later evolving into longitudinal analyses from 2016 onwards [15, 16, 17, 18, 19, 20]. These reports have driven advancements in minimally invasive techniques, with 123 research topics adopted between 2013 and 2024, 37 of which are directly related to minimally invasive surgery, including five focused on RAS.

In this first annual RAS report, we observed that operative times were relatively long across esophagectomy, gastrectomy, and proctectomy, whereas intraoperative blood loss and conversion rates to open surgery were notably low. Previous NCD‐based and international reports on conventional laparoscopic and thoracoscopic surgery have generally reported higher blood loss and higher conversion rates [15, 16, 17, 18, 19, 20, 21, 22, 23]. Although no direct contemporaneous statistical comparison with non‐RAS cohorts was conducted in this analysis, the low blood loss and very low conversion rates in the present RAS cohort appear favorable when interpreted in the context of these prior reports. Additionally, the lower BMI of the Japanese population, compared to Western populations, is likely a contributing factor to the extremely low rate of conversion to open surgery (less than 1%) [2, 21, 22, 23]. Although the R0 resection rate for esophageal cancer was lower and morbidity higher compared to those for rectal and gastric cancers, these outcomes were broadly consistent with those of traditional endoscopic approaches reported in guidelines and prior studies [24, 25, 26].

LPHS was longer in proportion to postoperative morbidity rate, but readmission rates within 30 days after surgery and postoperative mortality remained low, consistent with previous systematic reviews and large series of RAS in gastroenterological surgery [27, 28, 29]. These favorable outcomes are likely attributable to the high level of surgeon qualification and meticulous case selection [30, 31], despite RAS only recently received reimbursement.

A distinctive feature of the Japanese RAS introduction was the extremely high proportion of ESSQ‐qualified surgeons. In 2019, 98.9% of RAS procedures were performed by ESSQ‐certified surgeons, and RAS cases were concentrated in a relatively limited number of institutions. Previous NCD studies have shown that higher hospital volumes and structured training improve outcomes [15, 16, 17, 18, 19, 20]. Although a detailed volume–outcome analysis for RAS was not conducted here, these findings suggest that concentrating early RAS implementation in experienced centers with certified surgeons may have contributed to the low conversion and mortality rates. Future NCD analyses, incorporating multiple years of RAS data, will allow more robust stratification by facility volume and surgeon qualification to formally evaluate these relationships.

While the DVSS Xi model accounted for approximately two‐thirds of the robotic systems used in this report, recently approved robotic platforms by the PMDA now warrant evaluation to determine whether outcomes differ by system. Comparative effectiveness analyses among different robotic platforms, as well as between robotic and conventional minimally invasive approaches, will be important future directions.

## 5 Limitations

This study has several limitations. First, it is a single‐year cross‐sectional analysis, and temporal trends in RAS adoption and outcomes cannot yet be assessed. Second, postoperative mortality was defined as a composite of in‐hospital deaths within 90 days and deaths within 30 days after discharge, consistent with NCD standards but differing from international 30‐ and 90‐day definitions. Third, we did not directly compare RAS with non‐RAS cases or analyze volume–outcome relationships. These issues will be addressed in future multi‐year NCD analyses with larger datasets.

## 6 Conclusion

Although this report is a single‐year, cross‐sectional analysis, future cumulative reporting will allow for observation of RAS adoption trends over time. We anticipate that this initiative will enhance RAS quality, reduce regional disparities, optimize resource utilization, and foster medical innovation. This annual report serves as feedback for JSES members and aims to contribute to the ongoing advancement of robotic surgical medicine in Japan.

## Author Contributions

Study concept and design: Ichiro Takemasa, Ichiro Uyama, Masahiko Watanabe, Yoshiharu Sakai, and Yuko Kitagawa. Data acquisition and management: Ichiro Takemasa, Hiroyuki Yamamoto, Tatsuto Nishigori, Takeo Fujita, Tomoki Makino, Yusuke Taniyama, Masanori Terashima, Masanori Tokunaga, Takatoshi Matsuyama, Tomohiro Yamaguchi, Noriko Iwata, Hidetoshi Katsuno, Koichi Suda, Yusuke Kinugasa, Kazutaka Obama, Takashi Kamei. Data analysis and interpretation: Hiroyuki Yamamoto, Tatsuto Nishigori, Ichiro Takemasa. Drafting of the manuscript: Ichiro Takemasa, Hiroyuki Yamamoto, Tatsuto Nishigori.

## Conflicts of Interest

Yoshiharu Sakai is Editor in Chief of *Asian Journal of Endoscopic Surgery*. Masahiko Watanabe, Koichi Suda, Takashi Kamei, Kazutaka Obama, and Masanori Tokunaga are members of the editorial board of *Asian Journal of Endoscopic Surgery*. Hiroyuki Yamamoto is affiliated with the Department of Healthcare Quality Assessment at the University of Tokyo. The department is a social collaboration department supported by grants from the National Clinical Database, Intuitive Surgical Sarl, Johnson & Johnson K.K., and Nipro Co. The organizations were not involved in the planning, reporting, or interpretation of the present study. Other authors have no conflicts of interest.

## Supporting information


**Table S1:** Detail of esophagectomy.
**Table S2:** Lateral lymph node dissection in proctectomy.

## Data Availability

Research data are not shared.
